# The importance of morphological identification of African anopheline mosquitoes (Diptera: Culicidae) for malaria control programmes

**DOI:** 10.1186/s12936-018-2189-5

**Published:** 2018-01-22

**Authors:** Erica Erlank, Lizette L. Koekemoer, Maureen Coetzee

**Affiliations:** 1 Wits Research Institute for Malaria, MRC Collaborating Centre for Multidisciplinary Research on Malaria, School of Pathology, Faculty of Health Sciences, University of the Witwatersrand, Johannesburg, South Africa; 2 Centre for Emerging Zoonotic and Parasitic Diseases, National Institute for Communicable Diseases, Johannesburg, South Africa

**Keywords:** Anopheles, Africa, Identification, Morphology, PCR, Molecular

## Abstract

**Background:**

The correct identification of disease vectors is the first step towards implementing an effective control programme. Traditionally, for malaria control, this was based on the morphological differences observed in the adults and larvae between different mosquito species. However, the discovery of species complexes meant that genetic tools were needed to separate the sibling species and today there are standard molecular techniques that are used to identify the two major malaria vector groups of mosquitoes. On the assumption that species-diagnostic DNA polymerase chain reaction (PCR) assays are highly species-specific, experiments were conducted to investigate what would happen if non-vector species were randomly included in the molecular assays.

**Methods:**

Morphological keys for the Afrotropical Anophelinae were used to provide the a priori identifications. All mosquito specimens were then subjected to the standard PCR assays for members of the *Anopheles gambiae* complex and *Anopheles funestus* group.

**Results:**

One hundred and fifty mosquitoes belonging to 11 morphological species were processed. Three species (*Anopheles pretoriensis*, *Anopheles rufipes* and *Anopheles rhodesiensis*) amplified members of the *An. funestus* group and four species (*An. pretoriensis, An. rufipes, Anopheles listeri* and *Anopheles squamosus*) amplified members of the *An. gambiae* complex.

**Conclusions:**

Morphological identification of mosquitoes prior to PCR assays not only saves time and money in the laboratory, but also ensures that data received by malaria vector control programmes are useful for targeting the major vectors.

## Background

Malaria continues to be an ongoing problem in African countries south of the Sahara and although a lot has been achieved in the past 15 years, millions of people still remain at risk of contracting the parasite [1]. Africa provides a stable and ecologically diverse ecosystem and is home to the most efficient malaria vectors in the world [2–4], and is likely to remain so in the face of global climate change [5]. The major anopheline malaria vectors across sub-Saharan Africa are *Anopheles funestus* s.s. and three members of the *Anopheles gambiae* complex: *An. gambiae* s.s., *Anopheles coluzzii* and *Anopheles arabiensis* [2–4, 6–9]. There are, however, many additional species outside of these that play a role in malaria transmission within their geographic distribution, for example the *Anopheles moucheti* and *Anopheles nili* groups [3], and a host of secondary or incidental vectors [10–12]. Considering that the genus *Anopheles* contains over 500 species globally, of which only a few are considered important species for malaria transmission [2, 13], the morphological identification of species is crucial in order to target scarce resources for controlling the malaria vectors only.

Species groups and species complexes are common within the genus *Anopheles* [14] and this complicates vector control since not all species within a complex have similar behaviours or similar roles in malaria transmission [3, 4, 6]. In the *An. gambiae* complex, for example, species range from the non-vectors *Anopheles quadriannulatus* and *Anopheles amharicus* to minor vectors *Anopheles melas, Anopheles merus* and *Anopheles bwambae*, to the major vectors *An. gambiae, An. coluzzii* and *An. arabiensis* [4, 6, 9]. Genetic tools to identify the species have ranged from cross-mating wild mosquitoes with known laboratory colonies [15] to chromosomal banding patterns [16] and enzyme electrophoresis [17]. Today, the Scott et al. [18] molecular species-diagnostic PCR method is the gold standard for identifying members of the *An. gambiae* complex, and the five most common members of the *An. funestus* group are identified by the multiplex PCR assay of Koekemoer et al. [19]. However, prior to molecular analysis, accurate morphological identification is critical if correct interpretation of the results is to be used for vector control programme planning.

The purpose of the present study was to evaluate the impact of incorrect or no morphological identification of 150 specimens of 11 anopheline species from different African countries, by determining if these species could be misidentified using the standard vector-specific *An. gambiae* complex [18] and *An. funestus* group [19] PCR assays.

## Methods

### Mosquito collections

The species used in this study came from five African countries: Mali and Guinea in West Africa and Namibia, Botswana and South Africa in southern Africa. Collections were made between 2009 and 2017 by different entomological field teams on request and were made by sampling larvae, indoor resting catches and outdoor biting collections. Some were once off collections where field work was being conducted in other African countries, while other collections were made during routine surveillance around South African provinces. The inclusion of more specimens of the less abundant species in this study was limited due to methods of collection, species distribution and number of times that field sites were visited. Adults, larvae and pupae were brought back to Johannesburg, South Africa where further rearing of immatures to adults and processing of adult samples took place (Table 1). Adult mosquitoes were preserved in silica tubes for further morphological and molecular identification.

**Table 1:** Anopheline species collected from five African countries on which different species-diagnostic PCR analyses were conducted

Country	Province/site	GPS coordinates	Morphological ID	Molecular ID using *An. gambiae* complex PCR	Molecular ID using *An. funestus* group PCR	Molecular ID using *An. funestus*-like PCR	Molecular ID using *An. rivulorum*-like PCR
Botswana	Xakanaka	19°25′32.0″S 23°38′46.0″E	*An. wellcomei* group (n = 1)	–	–	–	–
Guinea Conakry	Siguiri	11°33′58.1″N 09°25′39.1″W	*An. rufipes* (n = 8)	–	*An. leesoni* (5)	–	–
Mali	Yanfolila	11°10′40.8″N 8°09′10.8″W	*An. rufipes* (n = 10)	–	*An. rivulorum* (1) *An. leesoni* (1)	–	–
Namibia	Katima Mulilo	17°29′48.1″S 24°15′51.5″E	*An. squamosus* (n = 2)	*An. gambiae* (2)	–	–	–
South Africa	Kwazulu-Natal	27°26′3.59**″**S 32°11′25.11**″**E	*An. rufipes* (n = 19)	–	–	–	–
	Gauteng	25°59′23.8″S 27°54′00.2″E	*An. crypticus* (n = 17)	–	–	–	–
			*An. marshallii* (n = 1)	–	–	–	–
	Mpumalanga	25°38′15.4″S 31°43′20.6″E	*An. coustani* (n = 6)	–	–	–	–
			*An. tenebrosus* (n = 1)	–	–	–	–
			*An. pretoriensis* (n = 20)	–	–	–	–
			*An. rufipes* (n = 9)	*An. gambiae/An. merus* (1)	–	–	–
			*An. maculipalpis* (n = 10)	–	–	–	–
	Limpopo	23°55′ 12.00″S 31°15′ 36.00″E	*An. maculipalpis* (n = 1)	–	–	–	–
			*An. rhodesiensis* (n = 1)	–	*An. leesoni* (1)	–	–
			*An. listeri* (n = 12)	*An. gambiae* (3) *An. merus* (2)	–	–	–
	Kruger National Park	23°59′18.2″S 31°33′16.9″E	*An. pretoriensis* (n = 26)	*An. arabiensis* (1) *An. gambiae* (3)	*An. funestus* (3) *An. vaneedeni* (1)	–	–
			*An. rufipes* (n = 6)	*An. arabiensis* (3) *An. gambiae* (2)		–	–

The total sample size (n) is indicated per morphological species according to each location and the number of specimens that amplified during the PCR analyses is shown for each assay

### Laboratory analyses

Adults were morphologically identified to species using the dichotomous key of Gillies and Coetzee [6]. DNA was extracted from a mosquito leg or wing using the prepGEM Insect DNA extraction kit (ZyGEM; E3BG6M; New Zealand). The amount of DNA used for each specimen was standard for the controls and specimens (1 µl). The DNA concentration that was present in the 1 µl of DNA extraction product was determined with the use of the NanoDrop One spectrophotometer (Thermo Scientific; Cat No. 13400518). A random sample (n = 42) produced an average of 39 ng/µl for the samples compared to the 44 ng/µl for the positive controls. Each specimen was then processed using PCR assays according to the standard protocols for the *An. gambiae* complex [18], *An. funestus* group [19], *An. funestus*-like [20] and *Anopheles rivulorum*-like [21]. Due to the fact that a number of non-specific amplification occurred during the latter two assays, only the first 111 samples were processed according to these protocols. Controls consisted of laboratory colony mosquitoes (*An. funestus* FUMOZ Mozambique; *An. coluzzii* NAG Nigeria; *An. arabiensis* AMAL South Africa; *An. merus* MAFUS South Africa and *An. quadriannulatus* SANGWE Zimbabwe) and two negative controls (one for DNA extraction and one for the PCR mix). DNA of a Malawian *An. funestus*-like specimen [20] was used for the *An. funestus*-like assay, and Kruger National Park specimens were used for the *An. rivulorum*-like control. The PCR product of each assay was electrophoresed on a 2.5% TAE agarose gel.

Gels were viewed with a Geldoc system and the amplicon sizes determined by comparing them with the base pair sizes of the DNA ladder. The base pair sizes were analysed with the QIAxcel Advanced system and DNA screening kit (2400) (Lot No. 154049015; Germany).

## Results

A total of 150 mosquito specimens belonging to 11 different anopheline species were analysed using four species-diagnostic PCR assays (Table 1). Five of the species amplified and produced diagnostic fragments for either *An. funestus* group or *An. gambiae* complex species or both (Table 2), but no amplification was observed for *Anopheles crypticus*, *Anopheles coustani*, *Anopheles tenebrosus*, *Anopheles wellcomei*, *Anopheles marshallii* or *Anopheles maculipalpis*. The assays for *An. funestus*-like and *An. rivulorum*-like produced non-specific amplifications where no distinct base pair size recognition could be made that was diagnostic for either of these two species.

**Table 2:** Morphological species that amplified to approximate base pair (bp) sizes of species within the *An. gambiae* complex and *An. funestus* group

Species complex	Expected band size (bp)	Morphological species that showed approximate amplification
*An. gambiae* complex		
*An. merus*	464	*An. rufipes*; *An. listeri*
*An. gambiae*	390	*An. squamosus*; *An. rufipes*; *An. listeri*; *An. pretoriensis*
*An. arabiensis*	315	*An. pretoriensis*; *An. rufipes*
*An. quadriannulatus*	153	
*An. funestus* group		
*An. vaneedeni*	587	*An. pretoriensis*
*An. funestus*	505	*An. pretoriensis*
*An. rivulorum*	411	*An. rufipes*
*An. parensis*	256	
*An. leesoni*	146	*An. rufipes*; *An. rhodesiensis*

Using the diagnostic *An. funestus* multiplex PCR assay, 8% (12/150) of the samples amplified. Seven of 52 *Anopheles rufipes* specimens had amplicon sizes corresponding to either *Anopheles leesoni* (n = 6) or *An. rivulorum* (n = 1), while the single *An. rhodesiensis* showed an amplicon size similar to *An. leesoni*. Only four out of 46 *Anopheles pretoriensis* specimens showed amplification during this assay—three produced a fragment size similar to *An. funestus* s.s. and one had a base pair size corresponding to that of *Anopheles vaneedeni.*

**Fig. 1:**
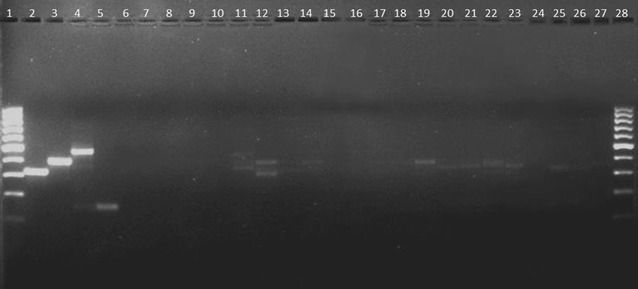
*Anopheles gambiae* species-diagnostic PCR gel electrophoresis showing the amplification by other anopheline species. Lanes 1 and 26: 100 bp DNA Ladder; lanes 2–5: positive controls for *An. arabiensis*; *An. gambiae; An. merus* and *An. quadriannulatus*; lanes 6, 7: negative controls for DNA extraction and PCR master mix; lane 10: *An. rufipes*; lanes 11–21: *An. listeri*; lanes 22, 23: *An. squamosus*; lanes 24, 25 contained no specimens

For the *An. gambiae* species-diagnostic PCR assay, 11.3% (17/150) amplified. Two *Anopheles squamosus* specimens showed amplicons similar to *An. gambiae* s.s. (Table 2). Seven out of 52 *An. rufipes* had amplifications similar to *An. gambiae* s.s. (n = 3), *An. arabiensis* (n = 3) and *An. merus* (n = 1). Five of the 12 *An. listeri* specimens showed fragments diagnostic for either *An. gambiae* s.s. (n = 3) or *An. merus* (n = 2). Four out of 46 *An. pretoriensis* samples amplified products similar in size to *An. arabiensis* (n = 1) and *An. gambiae* s.s. (n = 3). Some specimens also revealed non-specific amplification, most likely due to the fact that these protocols are not optimized for other anopheline species. Both amplification and non-amplification was present for specimens of the same species, irrespective of the amount of DNA that was present. Examples are shown in Fig. 1.

Interesting geographical variation was noted for *An. rufipes* in particular. Specimens from two West African countries, Guinea (Siguiri) and Mali (Yanfolila), had amplicons from the *An. funestus* species-diagnostic PCR assay but none for the *An. gambiae* species-diagnostic PCR assay. However, for South African sites it was the other way around with no amplification during the *An. funestus* species-diagnostic PCR assay and only amplicons produced by the *An. gambiae* assay in specimens from the Kruger National Park and surrounding areas in Mpumalanga.

## Discussion

As with other techniques used to identify mosquitoes, there are drawbacks to morphological species identification, such as when specimens have lost important external features of their anatomy (e.g. legs), a common occurrence when using collection methods such as CDC light traps where mosquitoes are damaged as they are sucked through the fan blades. In addition, the level of relevant skills to carry out the identifications may be inadequate or lacking.

A recent study carried out in eastern Zambia comparing morphological identifications with two molecular assays (COI mtDNA and ITS2 rDNA) [22] provides interesting insights. For example, of the 18 molecular species/forms they identified, 16 contained specimens that were morphologically identified as the *An. funestus* group and 12 as the *An. gambiae* complex. The molecular “*Anopheles coustani”* was being identified morphologically as both *An. funestus* and *An. gambiae* groups [22]. This variation either demonstrates a very low level of morphological skills, or there is a lot of variation in the molecular sequencing of individual mosquitoes. This latter is not unlikely given the amount of variation that was found in *An. gambiae* through the recently published 1000 Genome study [23]. Furthermore, sequence variability in the ITS2 region has been demonstrated previously between specimens of *An. rivulorum* from different countries [24] and this needs further investigation on a broad spectrum of species.

The present study showed that five of the 11 species amplified three members of the *An. gambiae* complex and four members of the *An. funestus* group. 30.8% of the *An. pretoriensis* gave amplicons of four different species, while 25% of the *An. rufipes* produced fragments of five species. But not every individual specimen of each species gave the same results, indicating considerable molecular variation. Some specimens also revealed non-specific amplification, most likely due to the fact that these protocols are not optimized for other anopheline species, thus possibly creating confusion in the routine analysis of the results.

It is well-known that almost every morphological taxon studied so far is a species complex. The best known are the *An. gambiae* complex and the *An. funestus* group based on various genetic techniques [6, 9, 25]. Others include *Anopheles coustani/crypticus* (chromosomes [26]), *Anopheles nili/ovengensis/carnevalei* (morphology, molecular [27, 28]) and *Anopheles marshallii/letabensis/hughi* (chromosomes [29, 30]). Complexes that still require formal morphological descriptions include *An. pharoensis* (chromosomes, 2 species [31]), *Anopheles longipalpis* (molecular, 2 species [32]) and *An. squamosus* (chromosomes, 5 species [Green and Hunt, unpublished data]). It is, therefore, not surprising when molecular studies show possible new species [22, 33], but in order to understand their importance in malaria transmission, these molecular forms need to be linked to iso-female lines that can be used to provide information on genetic variation within families and appropriate morphological descriptions. Such iso-female lines could also be used to produce reference sequence data for use in the development of accurate molecular species-diagnostic assays.

Morphological and molecular identification techniques should complement each other in order to intensify vector surveillance and our understanding of mosquito biodiversity [22, 34]. Morphological identification with the use of dichotomous keys forms the basis for the development of molecular techniques [35] and their subsequent use in surveillance or research [18, 19, 36, 37]. The above results indicate how important it is to carry out a priori morphological identification before using the standard molecular assays for the two major vector groups and if unusual species composition is reported, to initiate further investigations. Unfortunately, the loss of taxonomic expertise over the years has had a severe negative impact on describing mosquito biodiversity [14].

## Conclusions

This study showed that poor morphological identification cannot necessarily be detected and corrected during molecular PCR identification and can negatively affect vector surveillance since control interventions can likely be based on wrong identifications. Furthermore, processing mosquitoes for molecular identification is expensive and scarce resources should be limited to those specimens that require them. Malaria control programmes should continue to invest in capacity building for entomology teams and morphological training should be prioritized for entomological surveillance, particularly as more reports appear on secondary or incidental vectors playing a role in residual malaria transmission [10–12, 37, 38].
